# Parent‐of‐origin‐environment interactions in case‐parent triads with or without independent controls

**DOI:** 10.1111/ahg.12224

**Published:** 2017-11-02

**Authors:** Miriam Gjerdevik, Øystein A. Haaland, Julia Romanowska, Rolv T. Lie, Astanand Jugessur, Håkon K. Gjessing

**Affiliations:** ^1^ Department of Global Public Health and Primary Care University of Bergen Bergen Norway; ^2^ Department of Genetic Research and Bioinformatics Norwegian Institute of Public Health Oslo Norway; ^3^ Computional Biology Unit University of Bergen Bergen Norway; ^4^ Department of Health Registries Norwegian Institute of Public Health Oslo Norway; ^5^ Centre for Fertility and Health (CeFH) Norwegian Institute of Public Health Oslo Norway

**Keywords:** case–parent triad, gene–environment interaction, hybrid design, imprinting, parent‐of‐origin, power and sample size calculation, trios

## Abstract

With case–parent triad data, one can frequently deduce parent of origin of the child's alleles. This allows a parent‐of‐origin (PoO) effect to be estimated as the ratio of relative risks associated with the alleles inherited from the mother and the father, respectively. A possible cause of PoO effects is DNA methylation, leading to genomic imprinting. Because environmental exposures may influence methylation patterns, gene–environment interaction studies should be extended to allow for interactions between PoO effects and environmental exposures (i.e., PoOxE). One should thus search for loci where the environmental exposure modifies the PoO effect.

We have developed an extensive framework to analyze PoOxE effects in genome‐wide association studies (GWAS), based on complete or incomplete case–parent triads with or without independent control triads. The interaction approach is based on analyzing triads in each exposure stratum using maximum likelihood estimation in a log‐linear model. Interactions are then tested applying a Wald‐based posttest of parameters across strata. Our framework includes a complete setup for power calculations. We have implemented the models in the R software package Haplin.

To illustrate our PoOxE test, we applied the new methodology to top hits from our previous GWAS, assessing whether smoking during the periconceptional period modifies PoO effects on cleft palate only.

## 1 INTRODUCTION

A large number of human traits can be classified as complex, in the sense that they are assumed to be influenced by multiple genes and their interactions with environmental or behavioral factors (Pasaniuc & Price, 2016). Although thousands of genome‐wide association studies (GWAS) have been conducted since the turn of the millennium, for most complex traits the genetic variants identified thus far explain only a small fraction of the phenotypic variation attributed to genetic effects (Manolio et al., 2009). This has underscored the need to investigate disease mechanisms beyond simple genetic effects alone. One example is gene–environment interactions (GxE), where the genetic effects are modified by environmental exposures. For instance, Shi et al. (2007) have shown that maternal cigarette smoking in the periconceptional period can modify the association between single nucleotide polymorphisms (SNPs) and orofacial clefts.

With access to case–parent triad data, where an offspring and his/her parents have been genotyped, other genetic effects such as parent‐of‐origin (PoO) effects can be assessed. A PoO effect refers to the situation where the effect of a particular allele in the child depends on whether it is inherited from the mother or the father (Lawson, Cheverud, & Wolf, 2013; Connolly & Heron, 2014). For example, an allele might be protective when inherited from the mother but detrimental when inherited from the father. One example of a PoO effect is genomic imprinting, an epigenetic phenomenon where one of the inherited parental alleles is expressed whereas the other is silenced (Bartolomei & Tilghman, 1997; Reik & Walter, 2001). Although PoO effects are often used interchangeably with imprinting (Lawson et al., 2013), we here define PoO effects in statistical terms to mean an interaction effect; a PoO effect occurs if the phenotypic risk varies according to the parental origin of the variant allele.

In recent years, a growing number of studies have aimed to identify PoO and GxE effects separately for a wide range of diseases. However, it is reasonable to assume that the combined interaction effect (PoOxE effect) may also play an important role in complex traits. In our context, this means that the observed PoO effect may vary across environmental strata, which is plausible from a biologic perspective. A known cause of imprinting is DNA methylation in the germline. It is possible that maternal environmental exposures influencing methylation patterns might also influence the effects of maternally and paternally inherited alleles in unequal measures.

Conceivably, PoOxE effects may appear in different ways. The allele in question might increase risk only when transmitted from exposed mothers. A PoOxE effect may also be observed if the allele is protective to the child only when inherited from unexposed mothers but with no particular effect in the other situations. In principle, there might even be a “qualitative” interaction where the genetic effect is reversed. For instance, an allele might increase risk when inherited from exposed mothers and decrease risk when inherited from unexposed mothers, and concurrently decrease risk when inherited from exposed fathers and increase risk when inherited from unexposed fathers.

Another factor that needs to be controlled for in PoOxE models is the possible presence of maternal genetic effects. Maternal genetic effects occur when the genotype of the mother affects the phenotype of the child, regardless of the genetic material that has been transferred from mother to child (Connolly & Heron, 2014). Alleles carried by the mother may influence fetal development directly, for example, through maternal metabolic factors (Guilmatre & Sharp, 2012). This effect is distinct from PoO effects, in which we compare the effect of alleles *in the child*, depending on whether they were inherited from the mother or the father (Howey et al., 2015). Maternal genetic effects must therefore be estimated primarily from the nontransmitted allele of the mother, and appropriate models for PoOxE effects should allow maternal and PoO effects to be estimated simultaneously. Clearly, maternal effects are particularly important to studies of perinatal disorders.

Wang, Yu, Miller, Tang, and Perera (2011) previously introduced a test to screen for interactions between imprinted genes and environmental exposures. Still, there is a need to develop more general methods to investigate the joint effects of PoO and GxE (Lawson et al., 2013, p. 616). To address this gap in knowledge, we propose a novel approach that enables a full investigation of PoOxE effects. We develop our model for PoOxE within a flexible maximum‐likelihood framework based on log‐linear models (Gjessing & Lie, 2006; Skare et al., 2012; Jugessur, Skare, Harris, Lie, & Gjessing, 2012a), originally described in Wilcox, Weinberg, and Lie (1998), Weinberg, Wilcox, and Lie (1998), and Gjessing and Lie (2006). Our main study unit is the case‐parent triad, but it can be extended to include independent control children or control triads in a hybrid design (Weinberg & Umbach, 2005). Note that control triads are optional because the nontransmitted parental alleles implicitly serve as pseudocontrols (Knapp, Seuchter, & Baur, 1993; Schaid & Sommer, 1993; Cordell, Barratt, & Clayton, 2004; Cordell, 2004). Moreover, we use an expectation maximization (EM) algorithm (Dempster, Laird, & Rubin, 1977) to accommodate missing parents in mother–offspring or father–offspring dyads. A full implementation of our models is provided in Haplin, a flexible R package for genetic association analyses of single SNPs or haplotypes (Gjessing & Lie, 2006). The implementation uses parallel processing of SNPs, which makes GWAS analyses feasible. Haplin performs both testing and estimation of genetic effects. The framework also incorporates analyses of X‐chromosome SNPs in a natural way.

In statistical terms, PoO analyses are interaction analyses; the effect of an allele in the child may be modified by its parent of origin. In contrast, regular fetal‐effect analyses assume that the effect of an allele in the child is independent of whether it is transmitted from the mother or the father, that is, the effect is estimated without stratifying on parental origin. Higher sample sizes are thus required for PoO analyses to achieve the same statistical power as in regular fetal‐effect analyses. Accordingly, PoOxE analyses can be seen as second‐order interaction analyses. Hence, an even larger sample size is needed for a PoOxE analysis than for the corresponding PoO or GxE analysis to obtain the same statistical power. We therefore provide a thorough discussion of the power for PoOxE analyses and provide software to compute power for all relevant scenarios.

The article is structured as follows. In the Methods section, we first provide relevant background information and present the sampling and penetrance models. Next, we introduce our PoOxE test and derive the statistical methodology for single‐SNP analysis, and we also explain how PoOxE analyses can be carried out for SNPs on the X‐chromosome. We conclude the Methods section by presenting a previously published case triad study of orofacial clefts. In the Results section, we illustrate our PoOxE approach by using Haplin to analyze genetic triad data from the cleft study. We then assess the operating characteristics of the PoOxE test by investigating its power and attained significance level. The appendix includes a detailed discussion of PoOxE effects for haplotypes (Appendix A.1). Additionally, issues pertaining to sample size and power calculation are considered, and we present formulae and algorithms for our power computations (Appendix A.2). Haplin commands for estimating PoO, GxE and PoOxE effects on candidate genes are provided in the Supporting Information (S1). Statistical power calculations in Haplin are also covered in detail.

## 2 METHODS

### 2.1 Sampling and penetrance model

The likelihood model is based on a log‐linear model for the observed triad frequencies, conditional on the child being a case. Optionally, independent controls or control triads can be added to improve estimation of allele/haplotype frequencies. In this section, we describe the underlying sampling and penetrance model. A more detailed derivation of the log‐linear model is provided elsewhere (Gjessing & Lie, 2006).

We consider a single, multi‐allelic locus with *K* alleles *A*_1_, $A_{2},\ldots ,A_{K}$, with corresponding population allele frequencies *p*_1_, $p_{2},\ldots ,p_{K}$. The genotypes for the mother, father, and child are denoted by *M*, *F*, and *C*, respectively, and the full triad as (M,F,C) = ($A_{i}A_{j}$, $A_{k}A_{l}$, $A_{j}A_{l}$). For notational convenience, we assume that the second allele from the mother and the second allele from the father are transmitted to the child; that is, the full triad (M,F,C) can thus be described by the mating type (*M*, *F*) = ($A_{i}A_{j}$, $A_{k}A_{l}$).

The sampling model should describe the distribution of (M,F,C), conditional on the child being a case. If *D* denotes the event that the child is a case, Bayes' theorem allows our sampling model to be written as

(1) P(M,F,C|D)=P(D|M,F,C)P(M,F,C)/P(D).

The disease prevalence, P(D), cannot be observed directly from the case triad distribution and serves as a normalizing constant only. Assuming a population in Hardy–Weinberg equilibrium (HWE) with random mating and Mendelian transmission, we have

$$P\left(M,F,C\right)=P\left(A_{i}A_{j},A_{k}A_{l}\right)=p_{i}p_{j}p_{k}p_{l}.$$

Although the HWE assumption can be avoided using a more detailed parameterization (Weinberg et al., 1998; Gjessing & Lie, 2006), its inclusion in the model is convenient for computational efficiency and useful for reconstructing haplotypes. However, analyses should always include a strategy for checking large deviations from HWE because such deviations may be indicative of data issues. Top hits from a GWAS analysis should always be further investigated; Haplin performs a test for HWE on all SNPs.

The penetrance model, P(D|M,F,C), describes the probability of a child having the disease, conditional on the triad genotype. Assigning different effects to the alleles depending on parental origin, a penetrance model for PoO effects is

$$P\left(D|A_{i}A_{j},A_{k}A_{l}\right)=B\cdot {\text{RR}}_{M,j}{\text{RR}}_{F,l}{\text{RR}}_{jl}^{*},$$

where ${\text{RR}}_{M,j}$ and ${\text{RR}}_{F,j}$ are the risk increase (or decrease) associated with allele $A_{j}$, relative to the baseline risk level *B*, depending on whether the allele is transmitted from the mother or the father. The fraction ${\text{RR}}_{M,j}/{\text{RR}}_{F,j}$ is then a measure of the extent of the risk associated with allele $A_{j}$, depending on parental origin. The parameter ${\text{RR}}_{jl}^{*}$ is included to allow homozygous individuals to have a risk that deviates from what would be expected from a multiplicative model (e.g., dominant or recessive patterns). To incorporate this deviation, we have that ${\text{RR}}_{jl}^{*}={\text{RR}}_{j}^{*}$ when j=l and that ${\text{RR}}_{jl}^{*}=1$ when j≠l. Thus, if ${\text{RR}}_{j}^{*}=1$ for all *j*, the penetrance model is purely multiplicative. Note that *B* is typically associated with the reference allele and functions only as a normalizing constant. Moreover, this model also applies to multi‐allelic markers. The full sampling model (1) can then be parameterized as

$$\begin{array}{ccc} P(M,F,C|D) & = & P(A_{i}A_{j},A_{k}A_{l}|D)\\ & = & p_{i}p_{j}p_{k}p_{l}\cdot B\cdot {\text{RR}}_{M,j}{\text{RR}}_{F,l}{\text{RR}}_{jl}^{*}/P\left(D\right). \end{array}$$

Conditional on the child being a case, the triad type frequencies follow a multinomial distribution, and the parameters from the relevant sampling model are readily estimated by the method of maximum likelihood. The EM algorithm can be used to accommodate missing information, including reconstructing unknown haplotype phase from multiple markers. To ensure that the model is not overparameterized, one commonly sets RR=1 for a reference allele. Alternatively, population or reciprocal references can be used (Gjessing & Lie, 2006). Notice that throughout this article we assume a multiplicative dose–response relationship.

An important feature of the log‐linear model is the possibility to incorporate and adjust for maternal effects. Specifically, PoO and maternal genetic effects can be addressed simultaneously by the model

$$\begin{array}{ccc} P(D|A_{i}A_{j},A_{k}A_{l}) & = & B\cdot {\text{RR}}_{M,j}{\text{RR}}_{F,l}{\text{RR}}_{jl}^{*}\\ & & \times \;{\text{RR}}_{i}^{\left(M\right)}{\text{RR}}_{j}^{\left(M\right)}{\text{RR}}_{ij}^{(M)*}, \end{array}$$

where ${\text{RR}}_{i}^{\left(M\right)}$ is the relative risk associated with allele $A_{i}$ carried by the mother, and ${\text{RR}}_{ij}^{(M)*}$ is interpreted analogously to ${\text{RR}}_{ij}^{*}$. We thus assume that the maternal alleles have a multiplicative effect on top of the fetal alleles. Note specifically that in a combined model, the PoO effect is estimated essentially by contrasting allele frequencies of transmitted alleles, depending on parental origin, whereas the maternal effect is estimated by contrasting the frequencies of nontransmitted alleles in case mothers with that of nontransmitted alleles in case fathers.

Note that the PoO model requires information on parental origin, which is not available for ambiguous (uninformative) triads. However, the EM algorithm is implemented in our software and uses maximum likelihood to account for unknown parental origin in ambiguous triads. Additionally, it will account for missing information on individuals, such as when some triads are reduced to mother–child dyads due to missing data on the father. The basic model relates to a single multi‐allelic locus. In combination with the EM algorithm it extends directly to haplotypes over multiple loci by statistically reconstructing unknown haplotype phase (Gjessing & Lie, 2006).

### 2.2 Parent‐of‐origin‐environment interactions

Our PoOxE approach seamlessly integrates the PoO model with that of GxE. We therefore start by presenting and interpreting the PoO and GxE analyses separately, before combining them in the PoOxE test. The theory for PoOxE is here derived for a single SNP, but the extension to haplotypes is provided in Appendix A.1. We conclude the section by illustrating how PoOxE effects can be assessed on the X‐chromosome. Relevant Haplin commands for investigating PoO, GxE, and PoOxE effects are provided in S1.

For a single SNP, let ${\text{RR}}_{M}$ and ${\text{RR}}_{F}$ denote the relative risks associated with the variant allele (i.e., the nonreference allele) if it is inherited from the mother or from the father, respectively. We define the PoO effect as the relative risk ratio $\text{RRR}={\text{RR}}_{M}/{\text{RR}}_{F}$. This fraction is a measure of the magnitude of the risk associated with the allele under study, depending on whether it is maternally or paternally derived. A ratio larger than one indicates a higher risk when the variant allele is inherited from the mother versus the father. If it is equal to 1, the variant allele increases (or decreases) the risk by the same amount regardless of parental origin, and there is no PoO effect. For instance, if the variant allele doubles the risk of disease independently of parental origin, this is a standard fetal association; as such, it would have been identified in a traditional search for fetal gene effects. Note that one can assume a priori that, for instance, the paternal allele has no effect (i.e., ${\text{RR}}_{F}=1$) and try to detect a “pure” imprinting effect ${\text{RR}}_{M}$. This effect is, however, confounded with a standard fetal effect whenever the assumption ${\text{RR}}_{F}=1$ does not hold. Accordingly, we prefer to define our PoO test as a contrast between maternally and paternally derived allele risks.

Under the weak assumption of independence between exposure and child genotype conditional on parental mating type (Shi, Umbach, & Weinberg, 2010), interactions between genes and a categorical exposure variable can be incorporated into the log‐linear framework. Our GxE analyses fit the log‐linear model separately in each exposure stratum and consequently do not assume that allele frequencies are constant across strata. The model uses a Wald test to detect whether the relative risk estimates differ significantly across the exposure levels. In the situation of two exposure categories (1 = unexposed, 2 = exposed), we define ${\text{RR}}_{1}$ and ${\text{RR}}_{2}$ as the relative risks in the unexposed and exposed strata, respectively. The relative risk ratio $\text{RRR}={\text{RR}}_{2}/{\text{RR}}_{1}$ is a measure of the extent of the risk associated with the allele, depending on the exposure status of the case. For instance, a ratio larger than 1 implies that an exposed child carrying the variant allele has a higher risk than the unexposed child carrying the variant allele.

The PoO effect can be seen as a statistical interaction between the transmitted allele and its parental origin, whereas the GxE effect is an interaction between a main fetal effect with an external environment. It is thus natural to consider a PoOxE effect as a two‐way interaction that takes into account both parent of origin and environmental exposure in the same estimate. At a locus with two alleles and a dichotomous environmental exposure, the ratio

(2) $$\text{RRR}=\left({\text{RR}}_{M,2}/RR_{F,2}\right)/\left({\text{RR}}_{M,1}/RR_{F,1}\right)$$

is the PoO effect in the second stratum compared with the PoO effect in the first stratum. If RRR=1, it means that there may well be PoO effects, but that they, when measured on a multiplicative scale, are the same in both environmental strata. Similarly, since Eqn (2) may also be expressed as

$$\text{RRR}=\left({\text{RR}}_{M,2}/RR_{M,1}\right)/\left(RR_{F,2}/RR_{F,1}\right),$$

we will have RRR=1 if a GxE effect is the same for alleles of both parental origins. It is worth noting that the actual direction of an effect (i.e., RRR>1 or RRR<1) depends on which allele and exposure group are chosen as reference.

#### 2.2.1 The Wald test for interaction

In the log‐linear model, statistical inference is performed on log‐transformed relative risks and relative risk ratios. Thus, in the PoOxE situation, we would like to test the full interaction hypothesis

$$\beta_{M,1}-\beta_{F,1}=\beta_{M,2}-\beta_{F,2}=\cdots =\beta_{M,S}-\beta_{F,S},$$

where $\beta_{M,s}$ and $\beta_{F,s}$ are the log relative risks within stratum *s*, depending on whether the allele is derived from the mother or the father. Within each mutually exclusive exposure stratum, s=1,2,…,S, we calculate ${\hat{\beta }}_{s}={\hat{\beta }}_{M,s}-{\hat{\beta }}_{F,s}$, the difference between parental relative risks estimated on a log‐scale. From the asymptotic theory of log‐linear models (Christensen, 1997, Ch. 1 2.3), $\hat{\beta }$ follows approximately a multivariate normal distribution with mean ***β*** and variance–covariance matrix ***Σ***,

$$\begin{array}{c} \hat{\beta }=\left[\begin{array}{c} {\hat{\beta }}_{1}\\ {\hat{\beta }}_{2}\\ \vdots \\ {\hat{\beta }}_{S} \end{array}\right]∼\text{MVN}\left(\beta ,\Sigma \right). \end{array}$$

Because the strata are independent, the estimate of ***Σ*** is

Σ^=σ^120⋯00σ^22⋯0⋮⋮⋱⋮00⋯σ^S2= diag σ^12,σ^22,…,σ^S2,

where ${\hat{\sigma }}_{s}^{2}={\hat{\sigma }}_{M,s}^{2}+{\hat{\sigma }}_{F,s}^{2}-2{\hat{\rho }}_{M,F,s}{\hat{\sigma }}_{M,s}{\hat{\sigma }}_{F,s}$, with ${\hat{\rho }}_{M,F,s}$ being the correlation between ${\hat{\beta }}_{M,s}$ and ${\hat{\beta }}_{F,s}$ within stratum *s*.

The Wald test can then be used to conduct post‐hoc inference on the β parameters, based on the asymptotic normality (Agresti, 2013, Ch. 1.3). Let ***D*** be an appropriate r×S contrast matrix for the β parameters, with r≤S−1. It follows that asymptotically,

$$D\hat{\beta }∼\text{MVN}\left(D\beta ,\Sigma_{D}\right),$$

where ${\hat{\Sigma }}_{D}=D\hat{\Sigma }D^{T}$. The Wald test statistic is then

$$T={\left(D\hat{\beta }\right)}^{T}{\hat{\Sigma }}_{D}^{-1}\left(D\hat{\beta }\right).$$

Under the null hypothesis of Dβ=0, *T* has an approximate chi‐squared distribution with *r* degrees of freedom, $\chi^{2}\left(r\right)$.

In the PoOxE test, our null hypothesis can be seen as a test of all strata s=2,…,S against the first stratum s=1; that is, the test takes the form

$$\begin{array}{c} D\beta =\left[\begin{array}{ccccc} 1 & \;-1 & \;0 & \;\cdots & \;0\\ 1 & \;0 & \;-1 & \;\cdots & \;0\\ \vdots & \;\vdots & \;\vdots & \;\ddots & \;\vdots \\ 1 & \;0 & \;0 & \;\cdots & \;-1 \end{array}\right]\times \left[\begin{array}{c} \beta_{M,1}-\beta_{F,1}\\ \beta_{M,2}-\beta_{F,2}\\ \vdots \\ \beta_{M,S}-\beta_{F,S} \end{array}\right]=0. \end{array}$$

Hence, the Wald test statistic has an approximate χ^2^ distribution with r=S−1 degrees of freedom under the null hypothesis of no PoOxE effect. This is an overall test for any difference in PoO effects across strata when measured on a log risk scale.

Interactions with a continuous exposure variable can be incorporated in our framework by categorizing the variable into an appropriate number of categories and testing for a trend‐type association of the resulting ordinal variable. This approach is outlined for GxE effects in Skare et al. (2012), and a test for trend is included in Haplin.

#### 2.2.2 PoOxE analysis of X‐linked markers

Genetic association analyses of X‐linked markers are especially relevant if the prevalence of a complex trait differs systematically for males and females. Various penetrance models in Haplin address different causal scenarios that apply to an X‐linked disease locus. The models depend on the assumptions made regarding allele‐effects in males versus females, and might include sex‐specific baseline risks, shared or distinct relative risks for males and females, and X‐inactivation in females. A detailed description of parameterization models is provided in a previous study (Jugessur et al., 2012b). Haplin also allows for PoOxE analyses of X‐linked markers. Separate PoOxE analyses on males only are not possible; females are needed to obtain a contrast between maternally and paternally derived X‐chromosome alleles. However, fathers and male children contribute to estimating allele frequencies, and importantly, to facilitate haplotype reconstruction. Relevant Haplin commands for analyzing PoOxE effects on the X‐chromosome are provided in *S*1.

### 2.3 Case triad study: Cleft palate–only data analysis

Cleft palate only (CPO) is a common craniofacial birth defect in humans, occurring with (nonisolated) or without (isolated) other congenital anomalies or identifiable malformation syndromes. The prevalence rate for isolated CPO is 5 per 10,000 births worldwide (Mossey & Castilla, 2003). A wide array of genetic variants and environmental risk factors have been reported to increase the risk of CPO (Mossey, Little, Munger, Dixon, & Shaw, 2009; Dixon, Marazita, Beaty, & Murray, 2011; Rahimov, Jugessur, & Murray, 2012). However, as with many other complex traits, the genetic variants discovered so far only explain a minor fraction of the phenotypic variability. From our previously published GWAS (Beaty et al., 2010, 2011; Shi et al., 2012), the genotypes for 1575 individuals from 550 isolated CPO families were available, including 466 complete case–parent triads. These families were mainly of European and Asian ancestry, but a small number of families of other ethnicities were also present.

We considered three SNPs from the GWAS data to illustrate our PoOxE approach. On these SNPs, we conducted pooled analyses using all ethnicities, as well as separate analyses for Europeans only. The environmental factor was maternal cigarette smoking during the periconceptional period, that is, from 3 months before conception until 3 months into pregnancy, a window of exposure of 6 months in total. In the self‐administered questionnaire of the Norway Facial Clefts Study (https://www.niehs.nih.gov/research/atniehs/labs/epi/studies/ncl/index.cfm), this was evaluated as a simple yes/no response to ever having smoked during this period. The GWAS data set is available at the dbGAP database (http://www.ncbi.nlm.nih.gov/gap) under accession ID phs000094.v1.p1. Information on quality control and detailed characterizations of study participants and environmental exposure have been provided elsewhere (Haaland et al., 2017). Ethics approvals were obtained from the respective ethics committees for all the data in the cleft consortium. Background information on the study is provided in the original publication (Beaty et al., 2010).

## 3 RESULTS

### 3.1 Case triad study: Illustration of PoOxE data analysis

To illustrate our PoOxE test, we considered three SNPs from our GWAS data on CPO (Beaty et al., 2010, 2011; Shi et al., 2012). We only used top hits from previous studies, employing the same genetic triad data. Hence, the examples serve only as an illustration of our PoOxE test and not as independent replications of previous findings. Because our PoOxE approach integrates the PoO and GxE models, we start with examples of PoO effects (Table 1a) and GxE effects (Table 1b) before looking at the combined PoOxE effects (Table 1c).

**Table 1:** PoO, GxE and PoOxE effects for cleft palate‐only example SNPs

**a) rs7516430, *CHD1L*** 1				
Test effect	Stratum	${\text{RR}}_{M}$	${\text{RR}}_{F}$	${\text{RR}}_{M}/{\text{RR}}_{F}$
PoO effects*	${\text{RR}}_{\text{S}}$	1.79	0.52	3.42 (1.86, 6.15)
	${\text{RR}}_{\text{NS}}$	1.79	0.52	3.42 (1.86, 6.15)
	${\text{RR}}_{\text{S}}/{\text{RR}}_{\text{NS}}$	1 (–)	1 (–)	1 (–)
GxE effects**	${\text{RR}}_{\text{S}}$	1.22	1.22	1 (–)
	${\text{RR}}_{\text{NS}}$	1.06	1.06	1 (–)
	${\text{RR}}_{\text{S}}/{\text{RR}}_{\text{NS}}$	1.15 (0.51, 2.61)	1.15 (0.51, 2.61)	1 (–)
PoOxE effects	${\text{RR}}_{\text{S}}$	1.88	0.66	2.83 (0.90, 8.63)
	${\text{RR}}_{\text{NS}}$	1.76	0.48	3.68 (1.80, 7.37)
	${\text{RR}}_{\text{S}}/{\text{RR}}_{\text{NS}}$	1.07 (0.43, 2.69)	1.40 (0.40, 4.83)	0.77 (0.20, 2.91)
**b) rs470563, *ZNF236*** 2				
Test effect	Stratum	${\text{RR}}_{M}$	${\text{RR}}_{F}$	${\text{RR}}_{M}/{\text{RR}}_{F}$
PoO effects*	${\text{RR}}_{\text{S}}$	0.95	1.07	0.89 (0.67, 1.17)
	${\text{RR}}_{\text{NS}}$	0.95	1.07	0.89 (0.67, 1.17)
	${\text{RR}}_{\text{S}}/{\text{RR}}_{\text{NS}}$	1 (–)	1 (–)	1 (–)
GxE effects**	${\text{RR}}_{\text{S}}$	0.48	0.48	1 (–)
	${\text{RR}}_{\text{NS}}$	1.15	1.15	1 (–)
	${\text{RR}}_{\text{S}}/{\text{RR}}_{\text{NS}}$	0.42 (0.26, 0.68)	0.42 (0.26, 0.68)	1 (–)
PoOxE effects	${\text{RR}}_{\text{S}}$	0.44	0.52	0.86 (0.39, 1.87)
	${\text{RR}}_{\text{NS}}$	1.09	1.22	0.89 (0.66, 1.20)
	${\text{RR}}_{\text{S}}/{\text{RR}}_{\text{NS}}$	0.41 (0.21, 0.79)	0.42 (0.23, 0.80)	0.96 (0.41, 2.24)
**c) rs2964137, *ICE1*** 3				
Test effect	Stratum	${\text{RR}}_{M}$	${\text{RR}}_{F}$	${\text{RR}}_{M}/{\text{RR}}_{F}$
PoO effects*	${\text{RR}}_{\text{S}}$	1.42	1.06	1.34 (0.90, 1.97)
	${\text{RR}}_{\text{NS}}$	1.42	1.06	1.34 (0.90, 1.97)
	${\text{RR}}_{\text{S}}/{\text{RR}}_{\text{NS}}$	1 (–)	1 (–)	1 (–)
GxE effects**	${\text{RR}}_{\text{S}}$	1.16	1.16	1 (–)
	${\text{RR}}_{\text{NS}}$	1.25	1.25	1 (–)
	${\text{RR}}_{\text{S}}/{\text{RR}}_{\text{NS}}$	0.93 (0.54, 1.60)	0.93 (0.54, 1.60)	1 (–)
PoOxE effects	${\text{RR}}_{\text{S}}$	0.53	2.57	0.21 (0.09, 0.46)
	${\text{RR}}_{\text{NS}}$	1.88	0.85	2.22 (1.41, 3.43)
	${\text{RR}}_{\text{S}}/{\text{RR}}_{\text{NS}}$	0.28 (0.13, 0.58)	3.03 (1.45, 6.35)	0.09 (0.04, 0.24)

*PoO effects were estimated without stratifying on exposure. The rows corresponding to environmental strata are therefore equal by assumption.

**GxE effects were estimated without stratifying on parental origin. The columns related to ${\text{RR}}_{M}$ and ${\text{RR}}_{F}$ are therefore equal by assumption.

‐ The estimates are relative to the most frequent allele

‐ ${\text{RR}}_{M}$ and ${\text{RR}}_{F}$ are the relative risks depending on parental origin

‐ ${\text{RR}}_{\text{NS}}$ and ${\text{RR}}_{S}$ are the relative risks depending on exposure status (nonsmokers or smokers)

^1^Overall allele frequencies: A 0.88; T 0.12; Europeans only

^2^Overall allele frequencies: C 0.57; G 0.43; Whole sample

^3^Overall allele frequencies: G 0.52; C 0.48; Europeans only

The SNP rs7516430, located in the gene for “chromodomain helicase DNA binding protein 1‐like” or *CHD1L* on chromosome 1, had one of the most distinct signals in a previous PoO GWAS analysis of CPO by Shi et al. (2012). We re‐analyzed the data for this SNP on Europeans only, applying a Wald test. Table 1a (first row) presents the PoO estimates ${\text{RR}}_{M}$, ${\text{RR}}_{F}$ and $\text{RRR}={\text{RR}}_{M}/{\text{RR}}_{F}$. The most frequent allele, *A*, was used as reference. If allele *T* is inherited from the mother, it increases the risk of CPO. If, on the other hand, *T* is inherited from the father, the risk of CPO is nearly halved. As a result, RRR=3.42. There is a qualitative PoO effect with *P*‐value $5.6\times 10^{-5}$. Note that the PoO effects were estimated without stratifying on the exposure, smoking. Hence, by assumption, the estimates do not differ between strata. We still included the corresponding rows in the table to facilitate comparison with the following analyses. Table 1a also includes tests for GxE and PoOxE effects for this SNP (second and third row, respectively). However, no significant interactions were found.

The SNP rs470563 is associated with a higher risk of CPO in the presence of maternal smoking (Beaty et al., 2011). It is located in the gene “zinc finger protein 236” (*ZNF*236) on chromosome 18, and the re‐analyzed GxE results are presented in Table 1b (second row). Relative to allele *C*, allele *G* is associated with a decreased risk of CPO among smokers and an increased risk among nonsmokers. Consequently, RRR=0.42, and this qualitative effect has a *P*‐value of 4.5^−4^. It is important to note that although maternal smoking appears to be beneficial at first sight, this apparent risk‐reducing effect of smoking is contingent on the choice of reference allele. Switching the reference and variant allele inverts the estimated value of the RRR. Obviously, the main effect of smoking cannot be assessed from case‐triad designs alone, without independent controls. Therefore, the GxE RRR measures only how smoking *modifies* the estimated fetal genetic effects. For rs470563, we did not detect any significant PoO or PoOxE effects (Table 1b, first and third row, respectively). Note that the GxE effects were estimated without stratifying on parental origin. The columns in Table 1b, related to ${\text{RR}}_{M}$ and ${\text{RR}}_{F}$, are therefore equal by assumption.

In a separate study, we used the PoOxE test presented herein to perform a GWAS analysis of PoO interactions with maternal smoking and other exposures in Haplin (Haaland et al., 2017). The SNP rs2964137, located in the gene “interactor of little elongation complex ELL subunit 1” *(ICE1*), had one of the strongest signals in our search for PoOxE effects, and the PoO, GxE, and PoOxE results are shown in Table 1c. The risk estimates are relative to allele G, which is the most frequent. For this SNP, there is no evidence of a PoO effect independent of strata (first row) or of any GxE effect for fetal genes independent of parental origin (second row). Nevertheless, we found a qualitative PoOxE effect, RRR=0.09, with *P*‐value $6.5\times 10^{-7}$ (Table 1c, third row). The relative risk associated with allele C is nearly halved if derived from exposed mothers, and it is more than doubled if derived from exposed fathers. An opposite effect is seen in nonsmokers.

Haplin uses parallel processing of its analyses, and the run time of a GWAS analysis is therefore manageable. Our genome wide search for PoOxE effects was performed on Europeans only, comprising 762 individuals from 269 case families (mostly triads). Altogether 424,401 SNPs passed the quality controls and were included in our PoOxE analysis. We used eight CPU cores with 2.5 GHz per core, and the approximate run time of Haplin was 58 hours.

### 3.2 Operating characteristics and small sample behavior of the PoOxE test

We investigated the performance of our PoOxE test by evaluating its power in various settings. Power and sample size can be computed from the asymptotic variance–covariance structure underlying the Wald test; this approach is implemented in Haplin. The Haplin framework also includes a complete setup for power calculations through simulations, which is a robust way of checking software implementations, power, small‐sample behavior, and attained significance level. A detailed derivation of our asymptotic approximation formulae is given in Appendix A.2. Relevant example code for power calculations in Haplin is provided in S1.

**Figure 1:**
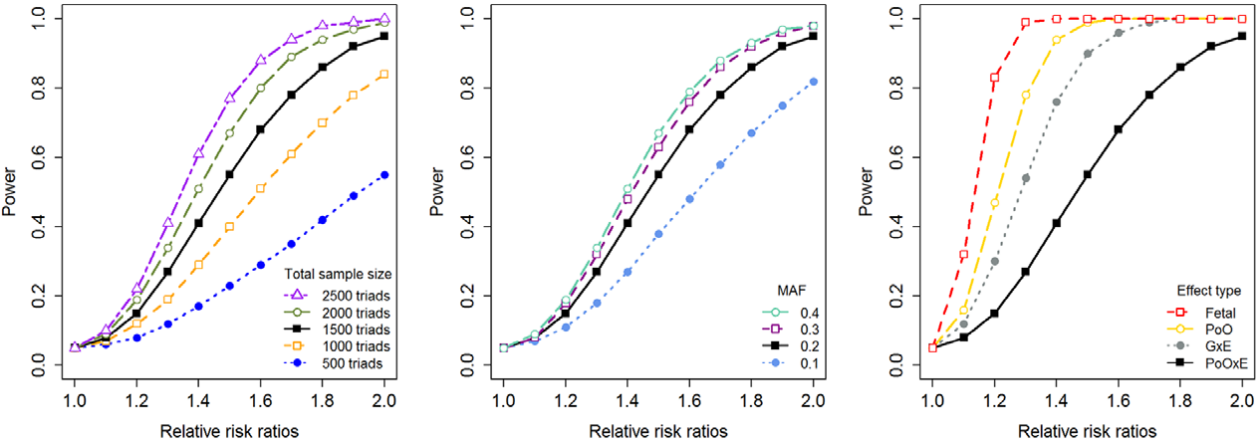
Single‐SNP power analysis for the PoOxE test for increasing relative risk ratios (increasing values of ${\text{RR}}_{M,2}$; ${\text{RR}}_{M,1}={\text{RR}}_{F,1}={\text{RR}}_{F,2}=1$) at the 0.05 nominal significance level. Equally sized exposure groups are assumed. Left panel: Increasing number of case–parent triads, and MAF=0.2; Middle panel: Increasing MAFs, and a total of 1500 case–parent triads; Right panel: Power comparison of the PoOxE, GxE (increasing values of ${\text{RR}}_{2}$; ${\text{RR}}_{1}=1$), PoO (increasing values of ${\text{RR}}_{M}$; ${\text{RR}}_{F}=1$), and fetal effect (increasing values of RR) tests, MAF=0.2, and a total of 1500 case–parent triads [Color figure can be viewed at wileyonlinelibrary.com]

We examined the power of the PoOxE test using the above‐mentioned asymptotic approximations. We first analyzed the power for a single SNP at the 5% nominal significance level. Power calculations for increasing relative risk ratios, RRRs, are shown in Figure 1. For simplicity, we set ${\text{RR}}_{M,1}$= ${\text{RR}}_{F,1}$ = ${\text{RR}}_{F,2}$ = 1 in all scenarios so that the value of RRR in Equation (2) is equal to the value of ${\text{RR}}_{M,2}$. Moreover, we assumed equally sized exposed and unexposed groups. The left panel of Figure 1 shows the statistical power for an increasing number of case–parent triads and a minor allele frequency (MAF) of 0.2. The black solid line is equal in all panels and is based on a total of 1500 case–parent triads, that is, 750 case–parent triads in both exposure categories. The middle panel depicts the power for increasing MAFs, using a total of 1500 case–parent triads. The right panel compares the power for various disease mechanisms (PoOxE, GxE, PoO, and fetal effects), using a total of 1500 case–parent triads and MAF = 0.2. Here, the fetal genetic effect is the direct risk associated with the child's allele, regardless of parent of origin or environmental exposures.

The power to detect PoOxE effects for a single SNP is sufficient for RRRs above 1.6–1.7 and a total sample size of 1500 case–parent triads with equally sized exposure groups. Nevertheless, larger sample sizes are needed if the MAF<0.2 or if the ratio of exposed versus unexposed is highly skewed (the latter result is not shown). Because the PoOxE test stratifies on both parent of origin and exposure, detecting a PoOxE effect requires a larger sample size than detecting a PoO effect or a GxE effect. Naturally, greatest power is achieved in a search for fetal effects.

**Figure 2:**
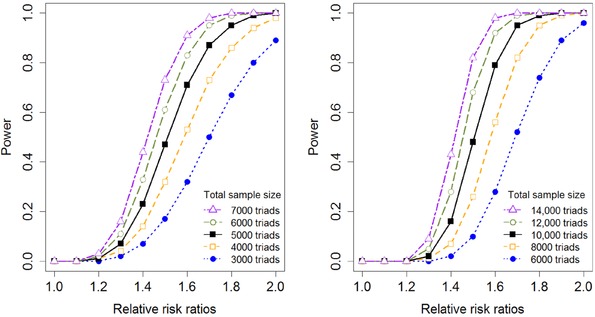
GWAS power analysis for the PoOxE test for increasing relative risk ratios (increasing values of ${\text{RR}}_{M,2}$; ${\text{RR}}_{M,1}={\text{RR}}_{F,1}={\text{RR}}_{F,2}=1$) and increasing number of case‐parent triads, assuming equally sized exposure groups and MAF=0.2. Left panel: Nominal significance level 10^−4^; right panel: Nominal significance level $5\times 10^{-8}$ [Color figure can be viewed at wileyonlinelibrary.com]

We also examined the power using nominal significance levels more relevant to GWAS settings. Figure 2 shows power analyses for increasing RRRs (i.e., increasing values of ${\text{RR}}_{M,2}$) with nominal significance levels 10^−4^ (left panel) and $5\times 10^{-8}$ (right panel). The power is demonstrated for an increasing number of case–parent triads using equally sized exposure groups and a MAF of 0.2. With a nominal significance level of 10^−4^, approximately 5000 case–parent triads are required to detect RRRs of 1.6–1.7 with 80% power. With a nominal significance level of $5\times 10^{-8}$, a sample size of 10,000 case‐parent triads suffices for RRRs above 1.6.

Our PoOxE test is asymptotically unbiased. However, the asymptotic approximations underlying log‐linear models may be suboptimal when the number of cases or controls is too small in one or more strata. When testing for GxE and PoOxE effects, one may occasionally encounter highly skewed exposure distributions. For example, in our CPO example, only 8 women of Asian ancestry answered “yes” to the question of maternal smoking during pregnancy, whereas the remaining 245 answered “no.” In such situations, the nominal significance level of the tests may be incorrect; the actual significance level is most easily assessed through simulations.

**Figure 3:**
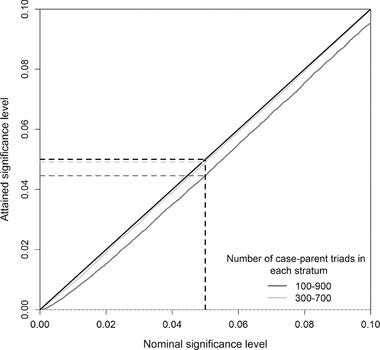
Simulated *P*‐values under the null hypothesis of no PoOxE effects based on 100,000 replications of data sets. The cumulative density plots compare the attained significance level with an expected uniform distribution under the null hypothesis (diagonal sloping line). A total of 1000 case–parent triads were divided into two exposure strata, and a MAF of 0.2 was assigned throughout. The distribution of case‐parent triads in each stratum was as follows: 100–900 (dark grey line) and 300–700 (light grey line). If no bias is present, the observed significance levels should equal the nominal level of 0.05 (black dashed lines). The dark and light grey dashed horizontal lines show the attained significance levels corresponding to the simulated scenarios

In Figure 3, cumulative density plots were used to examine the attained significance level of our PoOxE test. We obtained *P*‐values from 100,000 simulated data sets under the null hypothesis (${\text{RR}}_{M,1}$ = ${\text{RR}}_{M,2}$ = ${\text{RR}}_{F,1}$ = ${\text{RR}}_{F,2}$ = 1). The *P*‐values should be uniformly distributed when the null hypothesis is true. Hence, if no bias is present, the *P*‐values would fall close to the diagonal line. Throughout, a total of 1000 case–parent triads were divided into two exposure groups, and an MAF of 0.2 was assigned to both strata. Two scenarios were investigated according to the distribution of exposed and unexposed triads. In the first scenario (100–900), the smallest stratum comprised 100 case–parent triads. In the second scenario (300–700), the smallest stratum comprised 300 case–parent triads.

As expected, we observed a small bias for the PoOxE test when the number of cases in one exposure group was low, obtaining larger *P*‐values than expected. At the 0.05 nominal level, the attained significance level is 0.045 in the 100–900 setting. For lower significance levels, typically occurring in genome wide analyses, this bias might become substantial. Each exposure group should be large enough so that the asymptotic approximation of the estimator, $\hat{\beta }$, is sufficiently precise. Hence, the bias would be less pronounced for skewed exposure distributions at larger sample sizes (such as in a 1000–9000 setting). In other words, the unbalanced exposure design itself is not the cause of the observed deflation. The bias is negligible in the 300–700 setting, verifying that our PoOxE test attains the nominal significance level when the sample size of the smallest stratum increases.

## 4 CONCLUDING REMARKS

In this study, we have proposed a statistical method for detecting PoOxE effects. Postestimation in the log‐linear framework, incorporated into the Haplin software, allows us to combine the theory on PoO and GxE effects to test for the second‐order PoOxE effect. Although PoO and GxE studies abound, the combination has hardly been analyzed, in spite of its obvious biological relevance. Wang et al. (2011) proposed an interesting test to screen for interactions between imprinted genes and environmental exposures in a more restricted setting than our approach. Specifically, when testing for imprinted genes, Wang et al. assume that either the maternally or the paternally inherited allele is silenced so that only the other allele has an effect. This is in contrast to our PoO effect, which measures the difference between the effects of maternally and paternally derived alleles. Although the assumption of imprinted genes may increase testing power when it is true, it has the drawback of being more easily confused with ordinary fetal effects. For instance, if ${\text{RR}}_{M}={\text{RR}}_{F}=1.5>1$, this would trigger a test for imprinted genes but not for PoO.

Wang et al. (2011) use conditional logistic regression to analyze birth cohort designs with mother–offspring pairs. Our log‐linear framework is a general approach to the full hybrid design with complete or incomplete case triads possibly combined with control triads. We are therefore able to separate the effects of maternal alleles from the effect of maternally derived fetal alleles, which is particularly important in perinatal epidemiology, where the phenotype of the fetus can be influenced by either of the two sources (Hager, Cheverud, & Wolf, 2008). Additionally, our model provides a full maximum likelihood setup that allows us to estimate allele frequencies, haplotyping of multiple SNPs, and imputation of missing genotypes. Ambiguous (heterozygous) mother–offspring combinations need not be excluded as in the conditional logistic setup; they incorporate naturally into the model and provide data for the allele frequency estimation. Similarly, within the Haplin framework, PoOxE effects may also be detected on the X‐chromosome, where female offspring provide a contrast between maternally and paternally derived alleles; fathers and male offspring contribute to allele frequency estimation and precise haplotyping (Jugessur et al., 2012b). Finally, the data handling in Haplin enables a full genome‐wide screen for PoOxE effects.

Detailed study planning typically requires calculating the sample sizes needed to obtain sufficient power. Because statistical power depends on multiple factors including haplotype frequencies, penetrance model, and so on, published power tables for genetic studies are typically too restrictive, and software often covers only basic genetic models. As illustrated in S1, Haplin provides extensive power simulations, even covering the complex setup of PoOxE analyses. By entering the necessary parameters, the user can easily perform either “raw” simulations of power or use a very fast power calculation based on the asymptotic distribution of the parameter estimates.

In a GWAS analysis, the power to detect PoOxE effects is generally low. However, a candidate gene approach would reduce the complexity of multiple comparisons and enable a search for PoOxE effects when the sample size is limited. Specific environmental exposures that relate directly to the putative cause of the PoO effect of a candidate gene should be used in a PoOxE test. For example, one might assume that a detected PoOxE effect has a better chance of revealing a causal relationship involving genomic imprinting due to methylation than the standard PoO or GxE searches. A selection of relevant candidate genes might therefore be based on a GWAS screen for PoO or GxE effects.

Tracking the different etiologic mechanisms underlying complex diseases is crucial in improving diagnosis, prognosis, and prevention. The test for PoOxE effects and the comprehensive framework for assessing statistical power for genetic association analyses presented in this article are thus important contributions in advancing our understanding of the different etiologic mechanisms that underlie complex traits.

## 5 ELECTRONIC DATABASE INFORMATION

Haplin is implemented as a standard package in the statistical software ***R*** (R Core Team, 2016) and can be installed from the official R package archive, CRAN (https://cran.r‐project.org). Our website (http://folk.uib.no/gjessing/genetics/software/haplin) provides further information.

## ACKNOWLEDGEMENTS

The authors thank Prof. Ivar Heuch for his valuable comments.

## CONFLICT OF INTEREST

The authors declare that they have no competing interests.

## Supporting information


**Supporting Data S1**

**Table S1**: Values of ω^2^, the asymptotic variance of the log‐parameter for **a**) a complete case‐parent triad; and **b**) a complete case‐mother dyad.

## Figures and Tables

**Figure 4:**
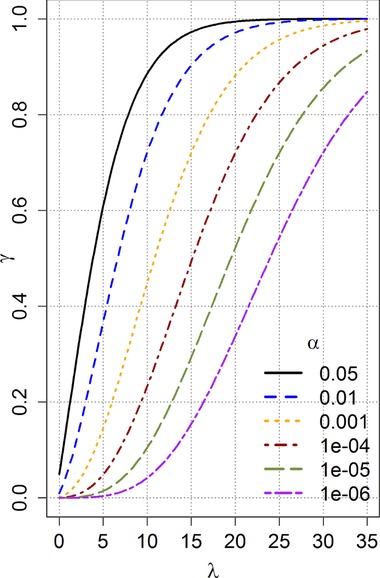
Power, γ, as a function of the noncentrality parameter, λ, for differing values of the nominal significance level, α. Here, $\lambda ={\left(\frac{\text{log(RR)}}{\omega /\sqrt{n}}\right)}^{2}$, where log(RR) is the log effect size, *n* is the number of case children, and ω is the asymptotic standard error of the log‐parameter. The number of degrees of freedom is equal to 1 [Color figure can be viewed at wileyonlinelibrary.com]

## APPENDIX A

### A.1

#### A.1 PoOxE effects in the haplotype situation

The majority of existing methods to investigate PoO and GxE effects are performed using a single‐marker approach in which each SNP is analyzed individually. However, haplotype analysis should enhance the possibility of “bracketing” a causal variant if the haplotype has a SNP on each side of the variant. The theory of PoOxE effects for the single‐marker setting can easily be extended to haplotypes. We here present a detailed derivation of the PoOxE test.

We assume a multiplicative dose–response effect and a reference haplotype approach. Without loss of generality, the first haplotype in arbitrary order is chosen as reference. Let *H* denote the number of haplotypes and *S* the number of independent exposure strata. We define ${\hat{\beta }}_{M,s}={\left[{\hat{\beta }}_{2,M,s},{\hat{\beta }}_{3,M,s},\ldots ,{\hat{\beta }}_{H,M,s}\right]}^{T}$ and ${\hat{\beta }}_{F,s}={\left[{\hat{\beta }}_{2,F,s},{\hat{\beta }}_{3,F,s},\ldots ,{\hat{\beta }}_{H,F,s}\right]}^{T}$, the relative risk estimates on a log‐scale for each haplotype within exposure stratum *s* (s=1,2,⋯,S), depending on parental origin. We calculate the difference ${\hat{\beta }}_{s}={\hat{\beta }}_{M,s}-{\hat{\beta }}_{F,s}$ and the corresponding asymptotic variance–covariance estimate

$$\begin{array}{c} {\hat{\Sigma }}_{s}=\left[\begin{array}{cc} {\hat{\Sigma }}_{M,s} & \;\;{\hat{\Sigma }}_{M,F,s}\\ {\hat{\Sigma }}_{M,F,s} & \;{\hat{\Sigma }}_{F,s} \end{array}\right], \end{array}$$

in which each element is a combined (H−1)×(H−1) variance–covariance matrix for haplotypes 2, 3, ..., *H*.

We would like to test the null hypothesis

$$\beta_{M,1}-\beta_{F,1}=\beta_{M,2}-\beta_{F,2}=\cdots =\beta_{M,S}-\beta_{F,S}.$$

This can be reformulated as

$$\begin{array}{c} D\beta =\left[\begin{array}{ccccc} I & \;-I & \;0 & \;\cdots & \;0\\ I & \;0 & \;-I & \;\cdots & \;0\\ \vdots & \;\vdots & \;\vdots & \;\ddots & \;\vdots \\ I & \;0 & \;0 & \;\cdots & \;-I \end{array}\right]\times \left[\begin{array}{c} \beta_{M,1}-\beta_{F,1}\\ \beta_{M,2}-\beta_{F,2}\\ \vdots \\ \beta_{M,S}-\beta_{F,S} \end{array}\right]=0. \end{array}$$

Here, ***I*** is the (H−1)×(H−1) identity matrix. From basic asymptotic theory of log‐linear models, we have that asymptotically

$$\hat{\beta }=\left[\begin{array}{c} {\hat{\beta }}_{1}\\ {\hat{\beta }}_{2}\\ \vdots \\ {\hat{\beta }}_{S} \end{array}\right]∼\mathrm{MVN}\left(\beta ,\Sigma \right),$$

where

Σ^= diag Σ^1,Σ^2,…,Σ^S.

Consequently, under the null hypothesis, the Wald statistic, $T={\left(D\hat{\beta }\right)}^{T}{\hat{\Sigma }}_{D}^{-1}\left(D\hat{\beta }\right)$, has an approximate χ^2^ distribution with (H−1)(S−1) degrees of freedom.

##### A.1.1 Haplotype example

Our Haplin framework allows a straightforward PoOxE analysis of haplotypes. As an illustration, we formed haplotypes by using one SNP on each side of the previously analyzed SNP rs2964137 in *ICE1* (i.e., rs2964447‐rs2964137‐rs6868526). We excluded haplotypes with frequencies below 1%, which left us with three haplotypes for our analysis. The results are displayed in Table 2, and the risk estimates are relative to the reference A‐C‐C haplotype. The first two SNPs are in strong linkage disequilibrium (*r* ^2^ = 0.996); the first SNP is therefore redundant and the same information can be obtained by using only the two last SNPs (*r* ^2^ = 0.427). Both the T‐G‐C and T‐G‐G haplotypes display PoOxE effects when analyzed separately against the reference, using the Wald test with one degree of freedom (*P*‐value = $2.1\times 10^{-5}$ and *P*‐value = $9.9\times 10^{-4}$). The PoOxE effect is stronger when both haplotypes are analyzed jointly, with 2 degrees of freedom (*P*‐value = $8.5\times 10^{-6}$). The separate relative risk estimates are fairly similar for the two haplotypes, indicating that the haplotype risks are driven by rs2964447 and rs2964137, which have the largest individual effect.

**Table 2:** PoOxE effects for cleft palate–only example haplotypes

rs2964447‐rs2964137‐rs6868526, *ICE1*				
Haplotype	Stratum	${\text{RR}}_{M}$	${\text{RR}}_{F}$	${\text{RR}}_{M}/{\text{RR}}_{F}$
T‐G‐C	${\text{RR}}_{\text{S}}$	1.99	0.49	4.04 (1.75, 9.25)
	${\text{RR}}_{\text{NS}}$	0.52	1.04	0.50 (0.31, 0.82)
	${\text{RR}}_{\text{S}}/{\text{RR}}_{\text{NS}}$	3.79 (1.74, 8.22)	0.47 (0.21, 1.05)	7.98 (3.07, 20.77)
T‐G‐G	${\text{RR}}_{\text{S}}$	1.30	0.24	5.35 (1.51, 18.19)
	${\text{RR}}_{\text{NS}}$	0.68	1.30	0.52 (0.29, 0.96)
	${\text{RR}}_{\text{S}}/{\text{RR}}_{\text{NS}}$	1.89 (0.70, 5.07)	0.19 (0.06, 0.62)	10.13 (2.55, 40.19)

‐Reference haplotype: A‐C‐C

‐Overall haplotype frequencies: A‐C‐C 0.48; T‐G‐C 0.36; T‐G‐G 0.16; Europeans only

‐${\text{RR}}_{M}$ and ${\text{RR}}_{F}$ are the relative risks depending on parental origin.

‐${\text{RR}}_{\text{NS}}$ and ${\text{RR}}_{S}$ are the relative risks depending on exposure status (nonsmokers or smokers)

The joint haplotype analysis loses some power compared to the single‐SNP analysis of rs2964137 due to haplotype reconstruction (*P*‐value $8.5\times 10^{-6}$ versus $6.5\cdot 10^{-7}$). Moreover, the Wald test statistic has 2 degrees of freedom. Nonetheless, we do not know a priori which approach, single‐marker or haplotype, will have the best likelihood of identifying an association.

#### A.2 Statistical power

The power of a genetic association analysis depends on numerous factors, such as significance level, allele/haplotype frequencies, effect size, and family design. A sample size calculation will typically involve computing the number of families needed to be genotyped to achieve a preset power for a given effect size. For instance, one might wish to achieve 80% power to detect a fetal effect of RR=2. The standard simulation approach to power calculations is the following. First, a sufficiently large number of data sets is simulated with appropriate parameter choices, such as effect size, sample size, family design, and so on. Then, the test is performed on each data set, and the power is the proportion of rejected null hypotheses. For a range of disease mechanisms, including PoO, GxE, and PoOxE effects, such power simulations are readily done in Haplin through the functions hapRun and hapPower. Relevant example code is provided in S1.

“Brute‐force” simulations are especially useful for small to moderate data sets. In such situations, only simulation studies can indicate the extent and direction of the possible bias. Nevertheless, both power and sample size can be computed much more efficiently directly from the asymptotic distributions underlying the Wald test. Such calculations have been implemented for a number of genetic effects in the Haplin function hapPowerAsymp. The principles behind the asymptotic calculations are standard; we will in the following paragraphs outline the specifics of our model implementations.

All tests described in this paper are performed as Wald tests, using the asymptotic normal distribution of the log‐scale parameters. In general, the power γ of the Wald test with level α is

(A.1) $$\gamma =1-F_{r,\lambda }\left(\chi_{\alpha }^{2}\left(r\right)\right),$$

where $\chi_{\alpha }^{2}\left(r\right)$ is the α quantile of the chi‐squared distribution with *r* degrees of freedom, $F_{r,\lambda }$ is the cumulative distribution function of a noncentral chi‐squared distribution $\chi^{2}\left(r,\lambda \right)$, and λ is the noncentrality parameter. To compute λ, consider first the simplest situation where we estimate a single effect, such as a fetal gene effect or a parent‐of‐origin effect, within a single stratum. Let *n* be the number of case children in the stratum. As *n* changes, we assume the composition of family structures within the stratum remains the same, relatively speaking. That is, we assume the ratio of control families to case families, the ratio of case mother–child dyads to complete case triads and so on, all remain the same. As before, we assume β=log(RR) is the log effect size in the stratum, and $\sigma^{\left(n\right)}$ is the standard error of $\hat{\beta }$ when estimated from all data in the stratum, with *n* case children. If the family structures are kept fixed as *n* increases, observe that $\sigma^{\left(n\right)}\approx \omega /\sqrt{n}$, where ω is the asymptotic standard error computed from the Fisher information in the maximum likelihood model. The value of ω is scaled to correspond to a sample with only one case child (n=1) in a stratum. For instance, in a setting with 200 case triad and 100 control triads, ω would, theoretically, correspond to a stratum with one case triad and half a control triad. Note that the ω parameter typically depends in a relatively complex way on the family design and allele/haplotype frequencies, and also on the effect sizes.

The noncentrality parameter λ is then the squared standardized log effect size (Agresti, 2013, Ch. 6.6), that is,

(A.2) $$\lambda ={\left(\frac{\text{log(RR)}}{\omega /\sqrt{n}}\right)}^{2}.$$

When the value of ω, corresponding to the appropriate model, has been determined, the power γ for a given sample size *n* is readily computed from Eqn (A.1), with r=1 and using the λ value computed from Eqn (A.2). Equivalently, for a given power γ, the necessary sample size can be computed by first finding the corresponding non‐centrality parameter λ from Eqn (A.1), and then solving Eqn (A.2) for *n* to obtain

(A.3) $$n=\lambda \omega^{2}/\log^{2}\text{(RR)}.$$

The relationship between γ and λ is illustrated in Figure 4 when r=1. Note that the lower significance levels are relevant in situations where multiple testing must be accounted for.

##### A.2.1 Sample size calculation for the PoO test

To ease the derivation of sample size estimation for the PoOxE test, we first illustrate the approach for our PoO test. When searching for PoO effects in a diallelic situation, the test statistic has one degree of freedom. Equations (A.1), (A.2), and (A.3) apply, with $\text{RR}={\text{RR}}_{M}/{\text{RR}}_{F}$. To facilitate power calculations “by hand” in simple situations, Table S1 provides the values of ω for selected PoO settings. Without loss of generality, in the following examples and derivations, we let the first allele in arbitrary order be the reference, with allele frequency 1−P. Note that if P>0.5, the reference allele is the minor allele.

Consider an example of sample size calculation for the PoO test. Let ${\text{RR}}_{M}=2$, ${\text{RR}}_{F}=1$, and P=0.1. From Table S1, we find that $\omega^{2}=19.5$. With level α=0.05 and desired power γ=80%, Figure 4 yields λ=7.85. Applying Eqn (A.3), we need roughly 320 case–parent triads or, equivalently, 344 case–mother dyads or 404 case–father dyads (the ω^2^ values for case–father dyads are not included in Table S1). Note that the values of ω^2^ depend not only on the ratio RR but also on the individual values of both ${\text{RR}}_{M}$ and ${\text{RR}}_{F}$. These calculations can be verified directly by power calculations in Haplin, as shown in S1.

Although a limited selection of values of ${\text{RR}}_{M}$ and ${\text{RR}}_{F}$ are included in Table S1, several symmetry relationships allow us to use the simple approach also in other scenarios. The power for testing PoO effects in case–parent triads for ${\text{RR}}_{M}=x$ and ${\text{RR}}_{F}=y$ is the same as when ${\text{RR}}_{M}=y$ and ${\text{RR}}_{F}=x$. Moreover, the power for testing PoO effects in triads if ${\text{RR}}_{M}=x$, ${\text{RR}}_{F}=y$, and P=p is identical to the power when ${\text{RR}}_{M}=1/x$, ${\text{RR}}_{F}=1/y$, and P=1−p. Finally, testing for PoO effects in case–mother dyads for ${\text{RR}}_{M}=x$, ${\text{RR}}_{F}=y$, and P=p is equivalent to testing for PoO effects in case–father dyads when ${\text{RR}}_{M}=1/y$, ${\text{RR}}_{F}=1/x$ and P=1−p.

##### A.2.2 Sample size calculation for the PoOxE test

We now consider two independent strata with sample size (number of case children) *n*_1_ and *n*_2_, respectively, where we want to compare ${\text{RR}}_{1}={\text{RR}}_{M,1}/{\text{RR}}_{F,1}$ in the first stratum with ${\text{RR}}_{2}={\text{RR}}_{M,2}/{\text{RR}}_{F,2}$ in the second stratum. The variance of $\beta =\left(\beta_{M,2}-\beta_{F,2}\right)-\left(\beta_{M,1}-\beta_{F,1}\right)$ is $\sigma_{1}^{2}+\sigma_{2}^{2}$, where $\sigma_{1}^{2}\approx \omega_{1}^{2}/n_{1}$ and $\sigma_{2}^{2}\approx \omega_{2}^{2}/n_{2}$ are the variances in the first and second stratum, respectively. The power to detect PoOxE effects is thus fully determined by the power to assess PoO effects in each stratum. Given power γ, significance level α, the stratum‐specific effects ${\text{RR}}_{1}$ and ${\text{RR}}_{2}$, and allele frequencies *P*_1_ and *P*_2_, as well as the ratio of sample sizes in the two strata, $\delta =n_{2}/n_{1}$, the PoOxE sample size calculation can be summarized in the following procedure:

1. Calculate $\omega_{1}^{2}$ and $\omega_{2}^{2}$ for the two exposure strata.
2. Calculate the sample size in the second stratum from the formula
  $$n_{2}=\frac{\lambda (\delta \omega_{1}^{2}+\omega_{2}^{2})}{{\text{log}}^{2}\left({\text{RR}}_{2}/{\text{RR}}_{1}\right)},$$
  where λ corresponds to the power γ.
3. Calculate the sample size in the first stratum, $n_{1}=n_{2}/\delta$.

Note that with two exposure strata, the number of degrees of freedom still equals one.

As an example, let ${\text{RR}}_{1}=1$, $P_{1}=0.3$, ${\text{RR}}_{2}=2.5$, and $P_{2}=0.1$, assuming $\text{RR}{}_{F}=1$ in both strata. For a given disease and environmental exposure, assume that it is reasonable to recruit twice as many case‐parent triads in the first stratum as in the second (i.e., δ=1/2). From Table S1a, we find that $\omega_{1}^{2}=$12.1 and $\omega_{2}^{2}=$18.6. Hence, it is sufficient to enroll approximately 460 triads in the first stratum and 230 triads in the second stratum to achieve 80% power at the 5% nominal significance level. The full power calculations for PoOxE effects have also been implemented in the Haplin function hapPowerAsymp.
