# Supplementary material for: Parent‐of‐origin‐environment interactions in case‐parent triads with or without independent controls
Source: Ann Hum Genet. 2017 Nov 2;82(2):60–73. doi: 10.1111/ahg.12224 (PMC5813215; doi:10.1111/ahg.12224)
Supplement: Supplementary file 1 — Supporting Data S1 [file AHG-82-60-s001.pdf]

S1. Haplin Commands

This section provides Haplin commands for analyzing PoO, GxE and PoOxE effects on candidate genes. We also show how a PoOxE analysis can be done on the X-chromosome. Relevant commands for power calculations are given, using both the asymptotic properties of the log-linear model and simulations. Haplin outputs are shown for selected examples. For a thorough description of the Haplin functions and their arguments, please refer our website at `http://folk.uib.no/gjessing/genetics/software/haplin`.

PoO, GxE and PoOxE Analyses on Candidate Genes

The fictive example file "data.dat" contains data on three SNPs in the native Haplin data format, although other data formats, including standard pedigree files, can easily be used for our analyses. Each line in the file represents a case-parent triad, with missing data on parents coded as NA. Information on maternal smoking is included as a covariate in the first column, followed by columns containing the genetic data.

Our PoO examples are analyzed in Haplin using commands similar to

```
res.PoO <- haplin(filename = "data.dat",
                markers = 2, n.vars = 1,
                design = "triad", poo = T,
                response = "mult", reference = "ref.cat",
                use.missing = T)
```

We here analyze the second SNP in the data set (`markers = 2`), and there is only one column in the data file to the left of the genetic data (`n.vars = 1`). The standard case-parent triad design without independent controls is specified by `design = "triad"`. The argument `poo = T` enables estimation of PoO effects. A multiplicative dose-response model is specified by `response = "mult"`;

reference = "ref.cat" chooses the most frequent allele/haplotype as reference. When use.missing is set to true, Haplin uses the EM algorithm to obtain risk estimates, accounting for incomplete triads. Note that both PoO and maternal risks, controlling for possible confounding with one another, are estimated simultaneously by including maternal = T. The most relevant output is tabulated by the command haptable(res.PoO).

The GxE effects are calculated by a two-step procedure. First, the genetic effects in each stratum of the environmental exposure are estimated using the function haplinStrat:

```
res.GxE <- haplinStrat(filename = "data.dat",
                markers = 2, n.vars = 1,
                strata = 1, design = "triad",
                poo = F, response = "mult",
                reference = "ref.cat", use.missing = T)
```

The exposure covariate is indicated by the argument strata. Second, the results from all strata are compared with the Wald test using the function gxe(res.GxE). Important output for each stratum is obtained by haptable(res.GxE).

Our PoOxE effects are estimated by similar commands to the GxE analyses. However, the argument poo must be set to true in haplinStrat. Haplin then computes the PoO effects within each stratum before contrasting the results through the function gxe. We here show an example of PoOxE analysis in the haplotype situation. The haplotypes are readily specified in Haplin through the argument markers, which in our case is formed by the first, second and third SNP in the data.

```
res.PoOxE <- haplinStrat(filename = "data.dat",
                markers = c(1,2,3), n.vars = 1,
                strata = 1, design = "triad",
                poo = T, response = "mult",
```

```
                reference = "ref.cat", use.missing = T)


gxe(res.PoOxE)
    gxe.test       chisq df          pval
1 haplo.freq   0.6910991  2 7.078313e-01
2        poo 23.3532786  2 8.489849e-06
```

For each test that is performed, the output shows the Wald chi-squared test value, degrees-of-freedom and the resulting p-value. Results for the haplotype frequency is always displayed in the first row. We are interested in the PoOxE results, which are here shown in the second row. Note that this is an overall test where the haplotypes are analyzed combined. Measures of relative risk ratios for each stratum and haplotype are obtained by `haptable(res.POOxE)`.

PoOxE Analysis on the X-Chromosome

PoOxE analyses on the X-chromosomes are carried out in a similar manner as for the autosomal markers, with the extension of three additional arguments. The argument `xchrom = T` enables analyses on X-linked markers. The `sex` argument indicates the data column containing the sex variable. In this example, we assume that a single allele in males has the same effect as a double allele dose in females. This corresponds to X-inactivation and is specified by `comb.sex = "double"`. However, if `comb.sex = "single"`, the effect of an allele in males is assumed to equal the effect of a single allele dose in females. PoOxE analyses on the X-chromosome can also be conducted for females only, as indicated by `comb.sex = "females"`.

```
res.PoOxE.xchrom <- haplinStrat(filename = "xchrom.dat",
                markers = 1, n.vars = 2, sex = 1,
                strata = 2, design = "triad",
                poo = T, xchrom = T,
```

```
                 comb.sex = "double", response = "mult",
                 reference = "ref.cat", use.missing = T)
```

Power Calculations

The asymptotic power can be computed directly in Haplin by the function `hapPowerAsymp`. The function extracts the asymptotic standard error of the estimated log-parameter and then uses the properties of the non-centrality parameter of the chi-squared distribution.

If the minor allele at a dichotomous locus is associated with a two-fold risk only when inherited from the mother, the asymptotic power for 200 case-parent triads is calculated using the command:

```
power.PoO <- hapPowerAsymp(nall = 2,
        cases = c(mfc=200), haplo.freq = c(0.9,0.1),
        RRcm = c(1,2), RRcf = c(1,1), RRstar = c(1,1))
```

The number of alleles at each locus is given by the vector `nall`. The allele frequencies are specified by the argument `haplo.freq`, and the corresponding relative risks are indicated by `RRcm` and `RRcf`, depending on parental origin. The family design is given by the arguments `cases` and `controls`. The nominal significance level equals 0.05 unless otherwise specified.

The power of GxE effects might be examined by a command similar to

```
power.GxE <- hapPowerAsymp(nall = 2, n.strata = 2,
        cases = list(c(mfc=400),c(mfc=200)),
        haplo.freq = c(0.9,0.1),
        RR = list(c(1,1),c(1,2.5)), RRstar = c(1,1))
```

The argument `n.strata` indicates the number of strata. Here, the number of case-parent triads varies between the two exposure categories, and the least frequent

allele is associated with disease only in the first stratum. The allele frequencies are the same in both strata. Extensions to several exposure levels are easily incorporated by modifying or expanding the appropriate arguments, e.g., `n.strata = 3`, `cases = list(c(mfc=400),c(mfc=200),c(mfc=100))` and `RR = list(c(1,1),c(1,2),c(1,3))`.

A power analysis for PoOxE interactions is achieved by combining the commands for PoO and GxE power calculations:

```
power.PoOxE <- hapPowerAsymp(nall = 2, n.strata = 2,
        cases = list(c(mfc=460),c(mfc=230)),
        haplo.freq = list(c(0.7,0.3),c(0.9,0.1)),
        RRcm = list(c(1,1),c(1,2.5)), RRcf = c(1,1),
        RRstar = c(1,1))


power.PoOxE
$haplo.power
  Haplotype RRcm.power RRcf.power RRcm_cf.power
1         1       0.94       0.05           0.8
2         2        ref        ref           ref
```

Here, there is only an effect of the maternally derived allele in the second stratum. The power to detect this change over strata is 94% (`RRcm.power`). The paternally derived allele has no effect in either stratum, so the corresponding power is 5%, i.e., equal to the nominal significance level. The actual PoOxE effect compares the two over strata, and thus has a somewhat lower power (`RRcm_cf.power`).

The statistical power for PoO, GxE and PoOxE interactions can also be computed through simulations. In Haplin, power simulations are carried out using a two-step procedure. First, `hapRun` is used to perform Haplin runs on simulated haplotype data, in which triad genotypes are generated from the multinomial distribution. The multinomial probabilities are calculated by listing all possible genotype

combinations in the triad format. It then employs the sampling model (equation 1 in the main text), with appropriate adjustments to the relevant effect situations. The second step feeds the simulation results to `hapPower`, and the power is subsequently computed by calculating the fraction of p-values less than the nominal significance level.

Provided that the large-sample properties of the log-linear model hold, the asymptotic power should be comparable with that obtained from simulations. The asymptotic power for the PoOxE analysis can be verified by the following commands:

```
sim.power.PoOxE <- hapRun(nall = c(2), n.strata = 2,
        cases = list(c(mfc=460),c(mfc=230)),
        haplo.freq = list(c(0.7,0.3),c(0.9,0.1)),
        RRcm = list(c(1,1),c(1,2.5)), RRcf = c(1,1),
        RRstar = c(1,1), poo = T,
        hapfunc = "haplinStrat", response = "mult",
        n.sim = 1000, cpus = 4)
hapPower(sim.power.PoOxE)
```

The arguments of `hapRun` are similar to those of `hapPowerAsymp` with a few exceptions. In addition to the arguments `RRcm` and `RRcf`, `poo` must be set to true in order to test for PoO effects in `hapRun`. Also, one needs to specify which haplin function to run, the response model and the number of simulations. The argument `cpus` speeds up computations by allowing parallel processing.
