# Supplementary material for: Parent‐of‐origin‐environment interactions in case‐parent triads with or without independent controls
Source: Ann Hum Genet. 2017 Nov 2;82(2):60–73. doi: 10.1111/ahg.12224 (PMC5813215; doi:10.1111/ahg.12224)
Supplement: Supplementary file 2 — Table S1: Values of ω2, the asymptotic variance of the log‐parameter for a) a complete case‐parent triad; and b) a complete case‐mother dyad. [file AHG-82-60-s002.pdf]

Table S1: Values of ω^2, the asymptotic variance of the log-parameter for **a)** a complete case-parent triad; and **b)** a complete case-mother dyad. The values are scaled to a sample of $n = 1$ triad or dyad, respectively.

a) Case-parent triad

RR_M	RR_F	$RR_M RR_F$	P 0.1	0.3	0.5	0.7	0.9
1	1	1	24.4	12.1	10.7	12.1	24.4
1	3/2	2/3	21.2	11.3	10.8	13.3	29.3
1	2	1/2	19.5	11.0	11.1	14.6	34.2
1	5/2	2/5	18.6	10.8	11.5	15.9	39.1
1	3	1/3	17.9	10.8	11.9	17.2	44.0
3/2	1	3/2	21.2	11.3	10.8	13.3	29.3
3/2	3/2	1	17.9	10.6	11.1	14.6	34.2
3/2	2	3/4	16.4	10.4	11.5	16.0	39.1
3/2	5/2	3/5	15.4	10.3	12.0	17.4	44.1
3/2	3	1/2	14.8	10.3	12.5	18.7	49.0
2	1	2	19.5	11.0	11.1	14.6	34.2
2	3/2	4/3	16.4	10.4	11.5	16.0	39.1
2	2	1	14.8	10.2	12.0	17.4	44.1
2	5/2	4/5	13.8	10.1	12.5	18.8	49.0
2	3	2/3	13.2	10.2	13.0	20.2	53.9
5/2	1	5/2	18.6	10.8	11.5	15.9	39.1
5/2	3/2	5/3	15.4	10.3	12.0	17.4	44.1
5/2	2	5/4	13.8	10.1	12.5	18.8	49.0
5/2	5/2	1	12.9	10.1	13.1	20.3	53.9
5/2	3	5/6	12.3	10.2	13.6	21.7	58.9
3	1	3	17.9	10.8	11.9	17.2	44.0
3	3/2	2	14.8	10.3	12.5	18.7	49.0
3	2	3/2	13.2	10.2	13.0	20.2	53.9
3	5/2	6/5	12.3	10.2	13.6	21.7	58.9
3	3	1	11.7	10.3	14.2	23.1	63.8

b) Case-mother dyad

RR_M	RR_F	$RR_M RR_F$	P 0.1	0.3	0.5	0.7	0.9
1	1	1	27.7	20.3	24.0	20.3	27.7
1	3/2	2/3	25.4	21.8	23.0	19.6	32.0
1	2	1/2	24.8	22.5	21.6	19.9	36.5
1	5/2	2/5	24.7	22.6	20.7	20.7	41.3
1	3	1/3	25.0	22.3	20.2	21.7	46.1
3/2	1	3/2	23.2	17.2	23.0	24.2	34.8
3/2	3/2	1	20.5	18.7	24.4	23.5	38.6
3/2	2	3/4	19.5	20.2	24.2	23.6	42.9
3/2	5/2	3/5	19.2	21.2	23.7	24.2	47.5
3/2	3	1/2	19.1	21.8	23.4	25.1	52.2
2	1	2	21.0	15.5	21.6	27.3	42.4
2	3/2	4/3	18.2	16.8	24.2	27.0	45.5
2	2	1	17.0	18.2	25.2	27.1	49.5
2	5/2	4/5	16.5	19.6	25.5	27.6	53.9
2	3	2/3	16.3	20.6	25.5	28.3	58.5
5/2	1	5/2	19.7	14.5	20.7	30.0	50.3
5/2	3/2	5/3	16.9	15.5	23.7	30.2	52.7
5/2	2	5/4	15.6	16.9	25.5	30.3	56.3
5/2	5/2	1	15.0	18.2	26.4	30.8	60.4
5/2	3	5/6	14.7	19.4	26.9	31.5	64.8
3	1	3	18.9	13.9	20.2	32.6	58.7
3	3/2	2	16.0	14.7	23.4	33.3	60.2
3	2	3/2	14.7	15.9	25.5	33.5	63.3
3	5/2	6/5	14.0	17.2	26.9	33.9	67.2
3	3	1	13.7	18.3	27.7	34.6	71.4

- P is the frequency of the non-reference allele
- RR_M and RR_F are the relative risks depending on parental origin
